# Combined use of CLP290 and bumetanide alleviates neuropathic pain and its mechanism after spinal cord injury in rats

**DOI:** 10.1111/cns.70045

**Published:** 2024-09-12

**Authors:** Yun‐zhu Pan, Zuliyaer Talifu, Xiao‐xin Wang, Han Ke, Chun‐jia Zhang, Xin Xu, De‐gang Yang, Yan Yu, Liang‐jie Du, Feng Gao, Jian‐Jun Li

**Affiliations:** ^1^ Rehabilitation Medicine Department, Beijing Hospital, National Center of Gerontology, Institute of Geriatric Medicine Chinese Academy of Medical Sciences Beijing China; ^2^ School of Rehabilitation Medicine Capital Medical University Beijing China; ^3^ Department of Spinal and Neural Functional Reconstruction China Rehabilitation Research Center Beijing China; ^4^ School of Rehabilitation Sciences and Engineering University of Health and Rehabilitation Sciences Qingdao China; ^5^ Chinese Institute of Rehabilitation Science Beijing China; ^6^ Center of Neural Injury and Repair Beijing Institute for Brain Disorders Beijing China; ^7^ Beijing Key Laboratory of Neural Injury and Rehabilitation Beijing China

**Keywords:** bumetanide, CLP290, KCC2, neuropathic pain, NKCC1, spinal cord injury

## Abstract

**Aim:**

We aimed to explore whether the combination of CLP290 and bumetanide maximally improves neuropathic pain following spinal cord injury (SCI) and its possible molecular mechanism.

**Methods:**

Rats were randomly divided into five groups: Sham, SCI + vehicle, SCI + CLP290, SCI + bumetanide, and SCI + combination (CLP290 + bumetanide). Drug administration commenced on the 7th day post‐injury (7 dpi) and continued for 14 days. All rats underwent behavioral assessments for 56 days to comprehensively evaluate the effects of interventions on mechanical pain, thermal pain, cold pain, motor function, and other relevant parameters. Electrophysiological assessments, immunoblotting, and immunofluorescence detection were performed at different timepoints post‐injury, with a specific focus on the expression and changes of KCC2 and NKCC1 proteins in the lumbar enlargement of the spinal cord.

**Results:**

CLP290 and bumetanide alleviated SCI‐associated hypersensitivity and locomotor function, with the combination providing enhanced recovery. The combined treatment group exhibited the most significant improvement in restoring Rate‐Dependent Depression (RDD) levels. In the combined treatment group and the two individual drug administration groups, the upregulation of potassium chloride cotransporter 2 (K^+^‐Cl^−^cotransporter 2, KCC2) expression and downregulation of sodium potassium chloride cotransporter 1 (Na^+^‐K^+^‐Cl^−^cotransporter 1, NKCC1) expression in the lumbar enlargement area resulted in a significant increase in the KCC2/NKCC1 ratio compared to the SCI + vehicle group, with the most pronounced improvement seen in the combined treatment group. Compared to the SCI + vehicle group, the SCI + bumetanide group showed no significant paw withdrawal thermal latency (PWTL) improvement at 21 and 35 dpi, but a notable enhancement at 56 dpi. In contrast, the SCI + CLP290 group significantly improved PWTL at 21 days, with non‐significant changes at 35 and 56 days. At 21 dpi, KCC2 expression was marginally higher in monotherapy groups versus SCI + vehicle, but not significantly. At 56 dpi, only the SCI + bumetanide group showed a significant difference in KCC2 expression compared to the control group.

**Conclusion:**

Combined application of CLP290 and bumetanide effectively increases the ratio of KCC2/NKCC1, restores RDD levels, enhances GABA_A_ receptor‐mediated inhibitory function in the spinal cord, and relieves neuropathic pain in SCI; Bumetanide significantly improves neuropathic pain in the long term, whereas CLP290 demonstrates a notable short‐term effect.

## Graphical abstract

- Combined application of CLP290 and Bumetanide can effectively increase the ratio of KCC2/NKCC1, restore RDD levels, enhance GABA_A_ receptor‐mediated inhibitory function in the spinal cord, and relieve neuropathic pain in SCI.
- Bumetanide significantly improves neuropathic pain in the long term, whereas CLP290 demonstrates a notable short‐term effect.

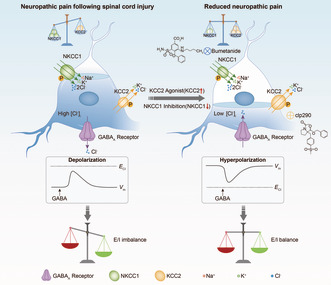

## 1 INTRODUCTION

The prevalence of neuropathic pain (NeP) after SCI ranges from 60% to 80%, and approximately two‐thirds of patients do not have appropriate treatment options,^1^ placing a greater burden on families and society.^2^, ^3^, ^4^, ^5^, ^6^ Traditional analgesic drugs include antidepressants, anticonvulsants, and opioids, which do not relieve pain completely and may have adverse side effects such as sedation, addiction, and motor function inhibition.^7^, ^8^, ^9^, ^10^, ^11^ An incomplete understanding of the molecular mechanisms underlying neuropathic pain has hampered the development of targeted interventions.

Currently, substantial evidence suggests that GABAergic inhibitory function is a key functional node in spinal nociceptive sensory processing and represents a potential target for therapeutic intervention.^12^, ^13^, ^14^ The polarity of GABA_A_R signaling depends on the precise regulation of intracellular chloride levels, which is determined by the two cation‐chloride cotransporters (CCCs) in the Solute Carrier Family 12(SLC12 family): KCC2 and NKCC1.^15^ KCC2 is the major Cl^−^ efflux cotransporter in neurons, whereas NKCC1 is the major Cl^−^ influx cotransporter. In healthy/mature conditions, relatively high KCC2 drives intracellular Cl^−^ concentrations ([Cl^−^]_i_) below its equilibrium potential (E_Cl_), thereby enhancing GABA_A_R‐mediated hyperpolarization and postsynaptic inhibition levels; whereas in pathological/immature conditions, relatively high NKCC1 drives [Cl^−^]_i_ above E_Cl_, enhancing GABA_A_R‐mediated depolarization and excitation.^16^, ^17^, ^18^, ^19^

Based on this knowledge, we hypothesized that up‐regulation of KCC2 or down‐regulation of NKCC1 may serve as molecular targets for treating neuropathic pain after SCI. Bumetanide is a well‐known loop diuretic and relatively specific NKCC1 inhibitor, which is effective in restoring low [Cl ^−^]_i_ and attenuating neuropathic pain and which has been tested in multiple clinical trials and has well‐established pharmacokinetic and pharmacodynamic properties with few side effects in humans.^20^, ^21^ CLP290 is a selective activator of KCC2, previously identified by Gagnon et al.^22^ We hypothesize that treatment with the combination of the KCC2 agonist CLP290 and NKCC1 inhibitor bumetanide may significantly improve neuropathic pain after SCI.

No studies have reported the effects of the combined use of KCC2 agonists and NKCC1 inhibitors in the treatment of neuropathic pain after SCI. Such molecular‐targeted drugs are usually used in non‐SCI models, specifically in models of peripheral nerve injury (PNI).^19^, ^23^, ^24^, ^25^ Therefore, in this study, we investigated the effects of the CLP290, bumetanide, and the combination of the two molecules on the improvement of neuropathic pain after SCI and determined the changes in KCC2 and NKCC1 expression and the possible molecular mechanism of their action in augmenting neuropathic pain.

## 2 METHODS

### 2.1 Experimental animals

Female Sprague–Dawley rats (8‐week‐old, weighing 220 ± 30 g) were obtained from Beijing Weitong Lihua Experimental Animal Technology Co., Ltd. (license number: SCXK [Beijing] 2012‐0001) and accommodated in the Animal Laboratory at the Chinese Academy of Rehabilitation Sciences. Rats were housed in standard cages maintained at room temperatures of 24–26°C with a light–dark cycle of 12 h. The Ethics Committee approved the experimental protocol and the use of animals in scientific experiments at Capital Medical University (Beijing, China, AEEI‐2022‐155).

### 2.2 Experimental design

**FIGURE 1:**
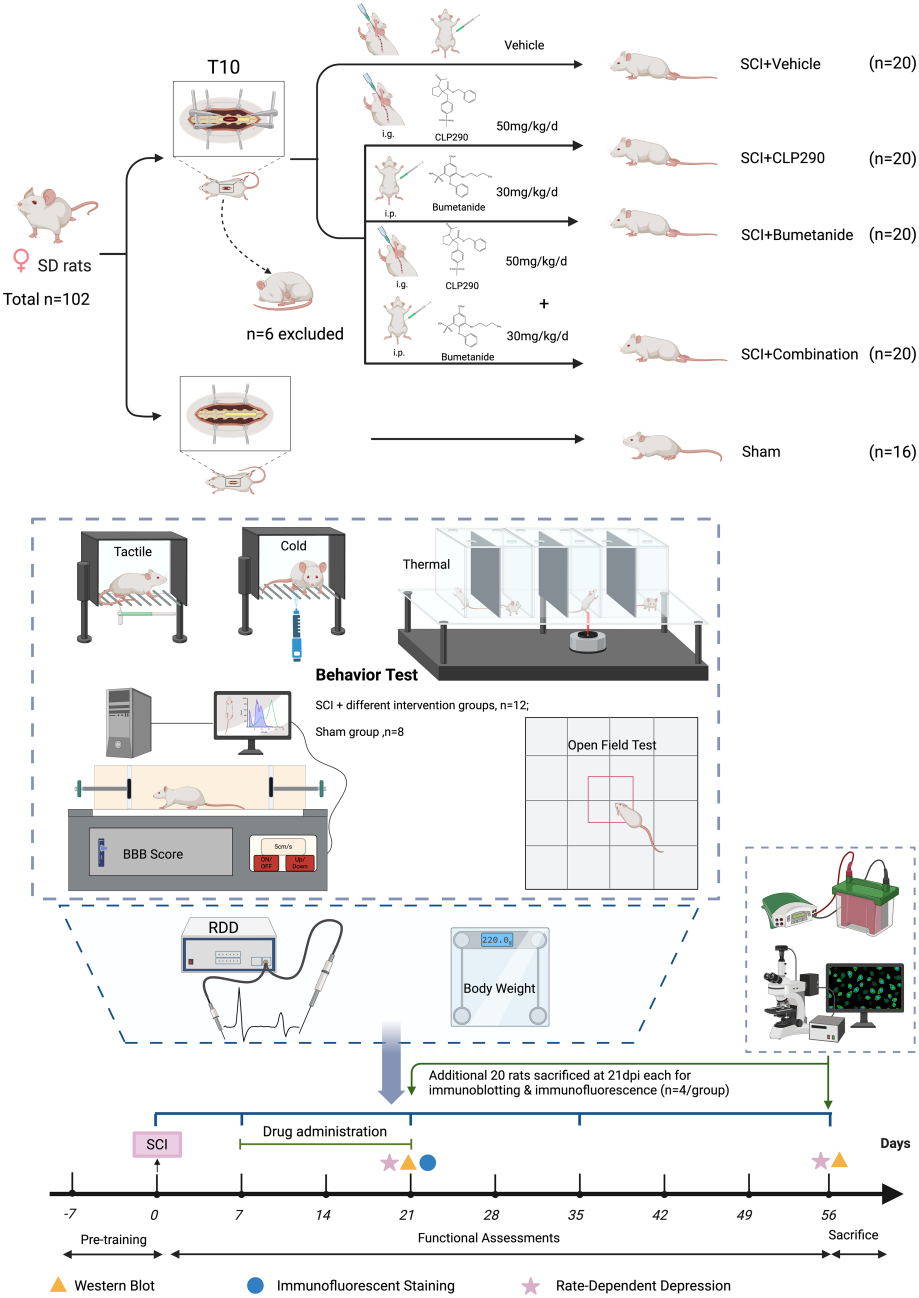
Experimental design. Rats were randomly divided into five groups: (1) Sham: Removal of the lamina of the T10 vertebrae without any other intervention; (2) SCI + vehicle: Moderate spinal cord contusion was performed at the T10 vertebral level and rats were treated with control vehicle; (3) SCI + CLP290: Administration of CLP290 at 50 mg/kg/day, intragastrically (i.g.) after SCI; (4) SCI + bumetanide: Administration of bumetanide at 30 mg/kg/day, intraperitoneally (i.p.) after SCI; and (5) SCI + combination: Combined administration of CLP290 at 50 mg/kg/day i.g. and bumetanide at 30 mg/kg/day i.p. after SCI. CLP290 was administered i.g. and bumetanide was administered i.p. once daily for 14 consecutive days from 7 dpi. A total of 102 adult female Sprague–Dawley rats (8‐week‐olds weighing 220 ± 30 g) were used in the study. Among them, 56 rats (Sham, *n* = 8; SCI + different intervention groups, *n* = 12) underwent behavioral assessments multiple times after surgery (0, 7, 21, 35, 56 dpi), and electrophysiological assessments were performed at 21 and 56 dpi (*n* = 8/group). Additionally, 20 rats were euthanized at 21 dpi for immunoblotting tests (*n* = 4/group), and another 20 were euthanized at 21 dpi for immunofluorescence assay (*n* = 4/group). At the end of the experiment, 20 rats used for behavioral measurements were euthanized at 56 dpi for immunoblotting (*n* = 4/group). A total of six rats were excluded: three rats died due to excessively low body weight and infection during the experiment, and three rats were excluded due to excessive asymmetry in the degree of paralysis in both lower extremities after the impact.

A total of 102 rats were utilized and were randomly allocated to five groups: Sham, SCI+ vehicle, SCI + CLP290, SCI+ bumetanide, and SCI+ combination (CLP290 + bumetanide). Among them, 56 rats underwent multiple behavioral and electrophysiological assessments following spinal cord contusion and were euthanized at 56 dpi for immunoblotting tests. Additionally, 20 rats were euthanized at 21 dpi for immunoblotting tests (*n* = 4 per group), and another 20 rats were euthanized at 21 dpi for immunofluorescence analysis (*n* = 4 per group). Six rats were excluded from the study due to mortality and spinal cord impact deviation. The experimental design is detailed in Figure 1 and its caption.

### 2.3 SCI model

The contusion injury method used in this study follows the protocol described in previous studies.^26^ Rats were anesthetized by inhalation of 2.5%–5% isoflurane. Briefly, laminectomy was performed at the T10 level. Then, both the rats in the SCI control group and those in the three treatment groups underwent spinal contusion injury at the T10 level. This injury was inflicted using a spinal cord impactor (IH‐0400 impactor, Precision Systems and Instrumentation, USA) with a force of 150‐kilodyne. Following the hit, a successfully established rat model of SCI was confirmed by the presence of spinal cord congestion, spasmodic swing of the tail, or retraction of both lower limbs. The animal was then sutured layer by layer and was kept warm until they were completely awake. Penicillin (12,000 μ/day) was injected after surgery to prevent infection. Bladders were manually emptied at least twice daily until spontaneous urination was restored. This procedure was adopted due to the relative ease of bladder expression and the lower risk of bladder infections and other complications, such as urethral blockages, observed in female rats compared to males, as documented in references.^27^, ^28^ Consequently, only female rats were utilized in the present study.

### 2.4 Drugs

The KCC2 agonist CLP290 (HY‐103023, MCE) was dissolved in a solution containing 50% PEG300 + 50% saline at a concentration of 50 mg/kg and administered intragastrically. The NKCC1 inhibitor bumetanide (HY‐17468, MCE) was prepared in 10 mg/mL saline solution containing 0.25% NaOH and administered intraperitoneally at a concentration of 30 mg/kg.^29^ The selection of the administration concentrations of CLP290 and bumetanide was based on the commonly used effective concentrations reported in previous literature.^22^, ^24^, ^30^, ^31^ Control vehicle refers to 10 mg/mL saline containing 50% PEG300 + 50% saline (i.g.) and 0.25% NaOH (i.p.). Treatment with these medications (or control vehicle) was initiated at 7 dpi, administered once daily, and continued for a duration of 14 days.

### 2.5 Assessment of mechanical allodynia

To assess mechanical allodynia, threshold levels to harmless mechanical stimulation were measured using 0.4–15 g of calibrated von Frey hairs.^32^ Briefly, eight Von Frey hairs (0.4, 0.6, 1, 2, 4, 6, 8, and 15 g) were selected. Each trial started with a Von Frey force of 2 g, which was applied vertically to the plantar surface of the rat hind‐paw. Abrupt withdrawal of the foot during stimulation or immediately after removal of the filament was recorded as a positive response. Once a positive or negative response was evoked, the next weaker or stronger filament was applied. This procedure was repeated until six stimuli following the first response were observed. The 50% paw withdrawal threshold (PWT) was calculated.^32^ Only rats with 50% PWT <4 g were selected and used for subsequent experiments.

### 2.6 Heat hyperalgesia

Paw withdrawal reflex latencies to noxious thermal stimuli were measured in rats using a plantar thermal algometer (Plantar Test; Ugo Basile, Italy).^33^ A movable focused beam of radiant heat was applied under the sole of one hind paw. When the rat retracted its paw from contact with the beam, a photocell turned off the heat, and the latency time was automatically recorded with a built‐in timer. The intensity of the radiant laser heat source below the glass was 50% (IR intensity). Both hind paws were measured three times, with measurements made 5 min apart on alternating paws, and the mean of all six measurements used as a composite score for each rat. To avoid tissue damage, the upper cut‐off limit was set at 25 s.

### 2.7 Cold allodynia

Cold allodynia was measured using an acetone test. Animals were placed on a wire mesh, and 100‐ul acetone was sprayed onto one hind paw using a syringe. Acetone was sprayed on one hind paw five times at approximately 2‐min intervals. Licking paws, shaking, or retracting legs were considered positive responses, and the total number of five positive responses was converted to a percent response frequency.^34^

### 2.8 Open field test

The Open field test is used to analyze behaviors such as locomotion, anxiety, and depression in rodents.^35^ For rats in this study, the experimental area consisted of a 50 × 50 × 50 cm polyvinyl chloride box, and movement of the rat into the center and peripheral areas of the box was monitored using a camera placed vertically in the center. The area of the center zone of the open field was 50% of the overall area. Spontaneous movement of each rat in the arena was recorded for 10 min with a highly sensitive camera, and movement tracks were analyzed using Cleversys TopScanlite software (Cleversys, VA, USA). The following behavioral parameters were analyzed: total number of central zone crossings, total horizontal movement distance, and mean horizontal movement velocity.

### 2.9 Rate‐dependent depression

The rats were anesthetized with 2% isopentane and placed on the operation table. Two 24‐g needle electrodes were inserted into the inner side of the lower limb ankle to stimulate the tibial nerve, and electrical signals were recorded at a stimulation wave width of 100 ms using an Electronic Stimulator (AD Instruments, New South Wales, Australia). The signals were amplified (×4000) and filtered (passband: 300 Hz to 6 kHz) using PowerLab software LabChart for analysis (Version 8.0, ADInstruments, New South Wales, Australia). To measure the RDD of the H reflex, we performed 20 consecutive stimuli at 0.1, 0.5, 1, 2, and 5 Hz, with a 2‐min interval between each series of stimuli. Fifteen stimuli were retained after each series of incentives to calculate their mean peak values. For statistical analysis, the average H wave value at 0.1 Hz was used as the baseline and was divided at 0.5, 1, 2, and 5 Hz by the value at 0.1 Hz to calculate the RDD of the H reflex.^36^

### 2.10 Basso, Beattie, and Bresnahan scales

Rat hindlimb motor function after SCI was evaluated using the BBB scales, with scores ranging from 0 (complete paralysis) to 21 (fully functional).^37^ Prior to each evaluation, rats were acclimated to the test environment for 15 min. The test area was a 90 cm diameter ring enclosed by a transparent barrier. Two independent researchers observed the activity of the rats over a period of 4 min.

### 2.11 Western blotting

The lumbar enlargement segments (L4‐L5 level, approximately 3 mm) of the rat spinal cord were collected while the rats were under deep anesthesia with sodium pentobarbital. Tissues were quickly frozen with liquid nitrogen and stored at −80°C for later testing. Tissue samples were lysed with RIPA buffer (P0013B, Beyotime), and the resulting homogenates were centrifuged at 12,000 rpm for 20 min at 4°C. Protein concentrations were determined using a BCA Protein Assay Kit (P0011, Beyotime) and adjusted to ensure consistent concentrations between groups. SDS‐PAGE electrophoresis (P0015L, Beyotime) was performed, and separated proteins were transferred to PVDF membranes (IPVH00010, Millipore). Membranes were probed overnight at 4°C with primary antibodies. The primary antibodies used were: KCC2 (07–432, Millipore; dilution 1:1000), NKCC1 (14,581, CST; dilution 1:1000). Then the membranes were washed three times before being incubated with secondary antibodies (1:3000 dilution). The membranes were then analyzed using chemiluminescence and gel image analysis, and the intensities of the selected bands were measured using Image J software.

### 2.12 Immunofluorescent staining

In brief, rats were anesthetized with sodium pentobarbital (40 mg/kg, i.p.), fixed on the operating table, and the skin and sternum were incised sequentially to expose the heart. Cardiac perfusion was performed first with 0.9% saline and later fixed with 4% paraformaldehyde solution. Following fixation, tissues from the lumbar enlargement (L2‐L6) of the rat spinal cord were collected. We further selected the most prominent segment of rat lumbar enlargement for sectioning and staining, which is located at the L4‐L5 segments of the spinal cord, aligning with the region we collected in our immunoblotting tests. Sections were separated by 300 μm so that each sensory neuron appeared in only one section. The mean fluorescence intensity of KCC2 (ab134300, Abcam, 1:200) and NKCC1 (85403, CST, 1:200) around sensory neurons in the superficial dorsal horn (SDH) (lamina I to IV) was determined by quantitative analysis of tissue sections by Strata Quest software (7.1).

### 2.13 Statistical analysis

Statistical analysis was performed using SPSS software (version 26.0; IBM Corp., Armonk, NY, USA). Measurement data were tested for normality using the Shapiro–Wilk test. Normally distributed measurement data were statistically described in the form of mean ± standard error on the mean (SEM); if not normally distributed, the median was used to represent central tendency, and the interquartile range indicated dispersion. Measurement data that met the normality and homogeneity of variance tests were analyzed using repeated measures analysis of variance (ANOVA) or one‐way ANOVA. Repeated measures ANOVA was used to analyze nociceptive thresholds, BBB scores, and RDD levels, followed by a Student Neuman–Keuls post‐hoc test. The statistical significance of Western blot and immunofluorescence intensity data was analyzed using one‐way ANOVA, followed by a Tukey post‐hoc test. Otherwise, comparisons were made using the non‐parametric Kruskal–Wallis H test, with Nemenyi/Dunn t used for post‐hoc testing. In all analyses, *p* < 0.05 was considered statistically significant.

## 3 RESULTS

### 3.1 The time‐course of KCC2 and NKCC1 protein expression following SCI in the lumbar enlargement of the spinal cord

We analyzed the expression of KCC2 and NKCC1 in the lumbar enlargement of the spinal cord in rats with spinal cord injury over time (Figure S1). The expression of KCC2 protein was significantly decreased at 3, 7, 21, 35, and 56 dpi compared to the sham group, reaching the lowest level at 7 dpi (3, 7, 21 dpi: *p* < 0.001; 35,56 dpi: *p* < 0.05). From 7 dpi, there was a trend of gradual recovery in KCC2 protein expression, but even at 56 dpi, there was still a significant difference in KCC2 expression levels compared to the sham group (*p* < 0.05) (Figure S1A). The NKCC1 protein was significantly increased at 7 dpi compared to the sham group (*p* < 0.01 vs. sham group). Although the expression of NKCC1 protein at 3, 21, 35, and 56 dpi was slightly higher than that in the sham group, it did not reach statistical significance (Figure S1B). Immunofluorescence staining results showed that at 21 dpi, KCC2 protein in the dorsal horn of the lumbar enlargement in the sham group rats exhibited high and intact expression intensity on neuronal cell membranes, while in the SCI group, the immunofluorescence intensity of KCC2 on neuronal cell membranes was lower and discontinuous, with punctate and patchy internalization of KCC2 visible in the cytoplasm. At the same time, the immunofluorescence intensity of NKCC1 on neuronal cell membranes was lower and discontinuous in the sham group, whereas in the SCI group, the expression of NKCC1 on neuronal cell membranes showed stronger and continuous immunofluorescence (Figure S1C).

### 3.2 CLP290 and bumetanide alleviated SCI‐associated hypersensitivity with the combination providing enhanced recovery

#### 3.2.1 Mechanical allodynia

**FIGURE 2:**
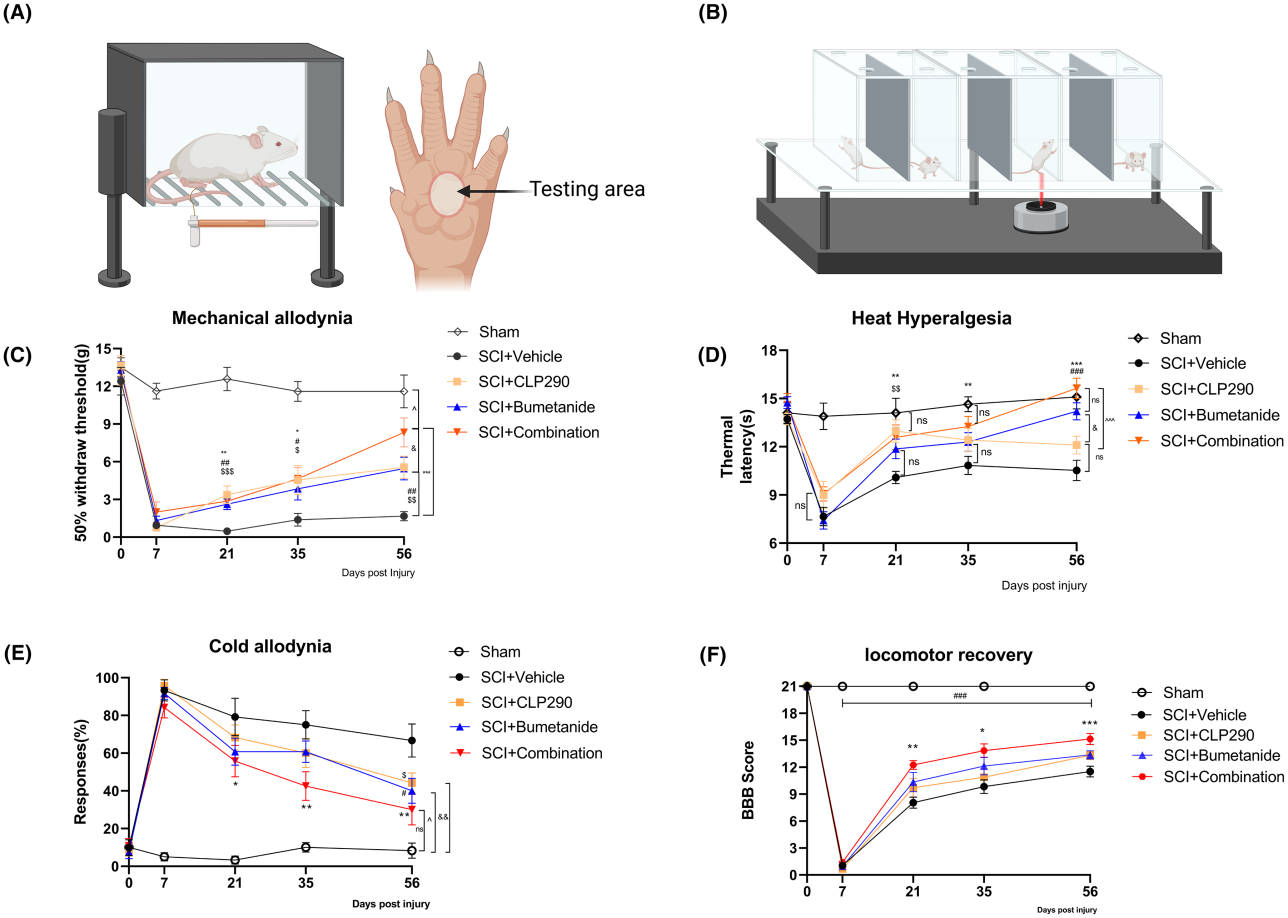
Effects of CLP290 and bumetanide, alone or in combination, on SCI‐associated hypersensitivity and locomotor function. (A) Von Frey evaluation model diagram. Rats were placed in transparent plastic cages (18 × 8 × 8 cm) on an elevated wire mesh surface and allowed to acclimate for approximately 30 min. To assess mechanical allodynia, threshold levels to harmless mechanical stimulation were measured using 0.4–15 g of calibrated von Frey hairs. (B) Von‐Frey fiber test area. The central plantar surface area of rat hind paw. (C) Effects of CLP290 and bumetanide, alone or combination, on SCI‐associated mechanical allodynia. *p < 0.05, **p < 0.01, ***p < 0.001 for SCI + combination versus SCI + vehicle; # *p* < 0.05, ##*p* < 0.01 for SCI + bumetanide versus SCI + vehicle; $*p* < 0.05, $$*p* < 0.01, $$$*p* < 0.001 for SCI + CLP290 versus SCI + vehicle; ^*p* < 0.05 for SCI + combination versus Sham; &*p* < 0.05 for SCI + combination versus SCI + CLP290 or bumetanide. (D) Effects of CLP290 and bumetanide, alone or combination, on SCI‐associated heat hyperalgesia. **p < 0.01, ***p < 0.001 for SCI + combination versus SCI + vehicle; ###*p* < 0.001 for SCI + bumetanide versus SCI + vehicle; $$*p* < 0.01 for SCI + CLP290 versus SCI + vehicle; ^*p* < 0.001 for SCI + combination versus SCI + CLP290; ns, *p* > 0.05. (E) Effects of CLP290 and bumetanide, alone or combination, on SCI‐associated cold allodynia. *p < 0.05, **p < 0.01 for SCI+ combination versus SCI + vehicle; #*p* < 0.05 for SCI+ bumetanide versus SCI+ vehicle; $*p* < 0.05 for SCI + CLP290 versus SCI+ vehicle; ^*p* < 0.05 for SCI+ bumetanide versus sham; &&*p* < 0.01 for SCI + CLP290 versus sham; ns, *p* > 0.05. (F) Effects of CLP290 and bumetanide, alone or in combination, on SCI‐associated locomotor function. *p < 0.05, **p < 0.01, ***p < 0.001 for SCI + combination vs SCI + vehicle; ###*p* < 0.001 for SCI+ CLP290, SCI+ bumetanide, SCI+ combination versus sham. Repeated measures two‐way ANOVA with Student Neuman–Keuls post‐hoc test. Values are mean ± SEM, Sham *n* = 8, SCI + different intervention groups *n* = 12.

After SCI, the decrease in paw withdrawal mechanical threshold (PWMT) was used to evaluate neuropathic pain at days 0, 7, 21, 35, and 56 (Figure 2C, Table S1). At 7 dpi, the PWMT of the other four SCI surgery groups was significantly lower than that of the sham group (*p* < 0.001). On postoperative days 21, 35, and 56, there was a significant improvement in the mechanical threshold of the three treatment groups compared to the SCI + vehicle group (*p* < 0.05). The combination group showed no significant difference in PWMT compared to the other two drug groups at 21‐ and 35‐days post‐surgery (*p* > 0.05). However, at 56 dpi, the PWMT of the combination drug group was significantly higher than that of the other two drug groups (vs. SCI + CLP290, *p* = 0.032; vs. SCI + bumetanide, *p* = 0.026). These data indicate that the combination of CLP290 and bumetanide maximizes in relieving mechanical allodynia than either drug used alone.

#### 3.2.2 Heat hyperalgesia

At 7 dpi, the four SCI surgery groups had a significantly decreased paw withdrawal thermal latency (PWTL) compared to the sham group (*p* < 0.001) (Figure 2D, Table S2). The SCI + bumetanide group showed no significant improvement in PWTL at 21 and 35 dpi compared to the SCI + vehicle group (*p* > 0.05). However, at 56 dpi, there was a significant improvement (*p* < 0.001). The SCI + CLP290 group showed significant improvement in PWTL at 21 days compared to the SCI + vehicle group (*p* < 0.01), and this improvement did not reach statistical significance at 35 and 56 days (*p* > 0.05). The combined use of CLP290 and bumetanide significantly improved PWTL when compared to the SCI + vehicle control group throughout the experiment (21 dpi, *p* < 0.01; 35 dpi, *p* < 0.01; 56 dpi, *p* < 0.001), and there was no significant difference in the PWTL when compared to the sham group at 21, 35, and 56 dpi (*p* > 0.05). Therefore, it is evident that the combined use of CLP290 and bumetanide maximizes the improvement in thermal hypersensitivity.

#### 3.2.3 Cold allodynia

At 21, 35, and 56 days after surgery, the SCI + combination group showed significantly lower acetone response compared to the SCI + vehicle group (21 dpi, *p* = 0.038; 35 dpi, *p* = 0.01; 56 dpi, *p* = 0.01), and there was no significant difference in acetone response between the SCI + combination group and the sham group at 56 dpi (*p* > 0.05) (Figure 2E, Table S3). At 21 and 35 dpi, the acetone response in the SCI + bumetanide and SCI + CLP290 groups was slightly lower than that in the SCI + vehicle group, with no statistically significant difference (*p* > 0.05), but had significantly lower acetone responses at 56 dpi (*p* = 0.028, vs. SCI + CLP290; *p* = 0.01, vs. SCI + bumetanide). Therefore, all three administration treatment groups improved cold hyperalgesia to varying degrees, with the combination group showing the greatest improvement.

### 3.3 Effects of CLP290 and bumetanide, alone or in combination, on SCI‐associated locomotor function

BBB scores were used to evaluate hindlimb motor function after SCI (Figure 2F). From 7 dpi until the end of the experiment, the BBB scores for the four SCI groups were significantly lower than those of the sham group (*p* < 0.001). While the SCI + bumetanide and SCI + CLP290 groups had slightly improved BBB scores at 21, 35, and 56 dpi, there were no significant differences (*p* > 0.05) compared to the SCI + vehicle group. However, the BBB score of the SCI + combination group was significantly higher than that of the SCI + vehicle group at 21, 35, and 56 dpi (21 dpi, *p* = 0.005; 35 dpi, *p* = 0.012; 56 dpi, *p* < 0.001). The study also investigated temporal changes in body weight of rats within experimental groups (as shown in Figure 2A) and compared the differences between groups (as shown in Figure 2B). Specific body weight values can also be found in Table S4. These findings suggest that the combination treatment maximally improved BBB scores in rats following SCI.

### 3.4 Effects of CLP290 and bumetanide, Alone or in Combination, on improving anxiety, depression, and activity level following SCI

**FIGURE 3:**
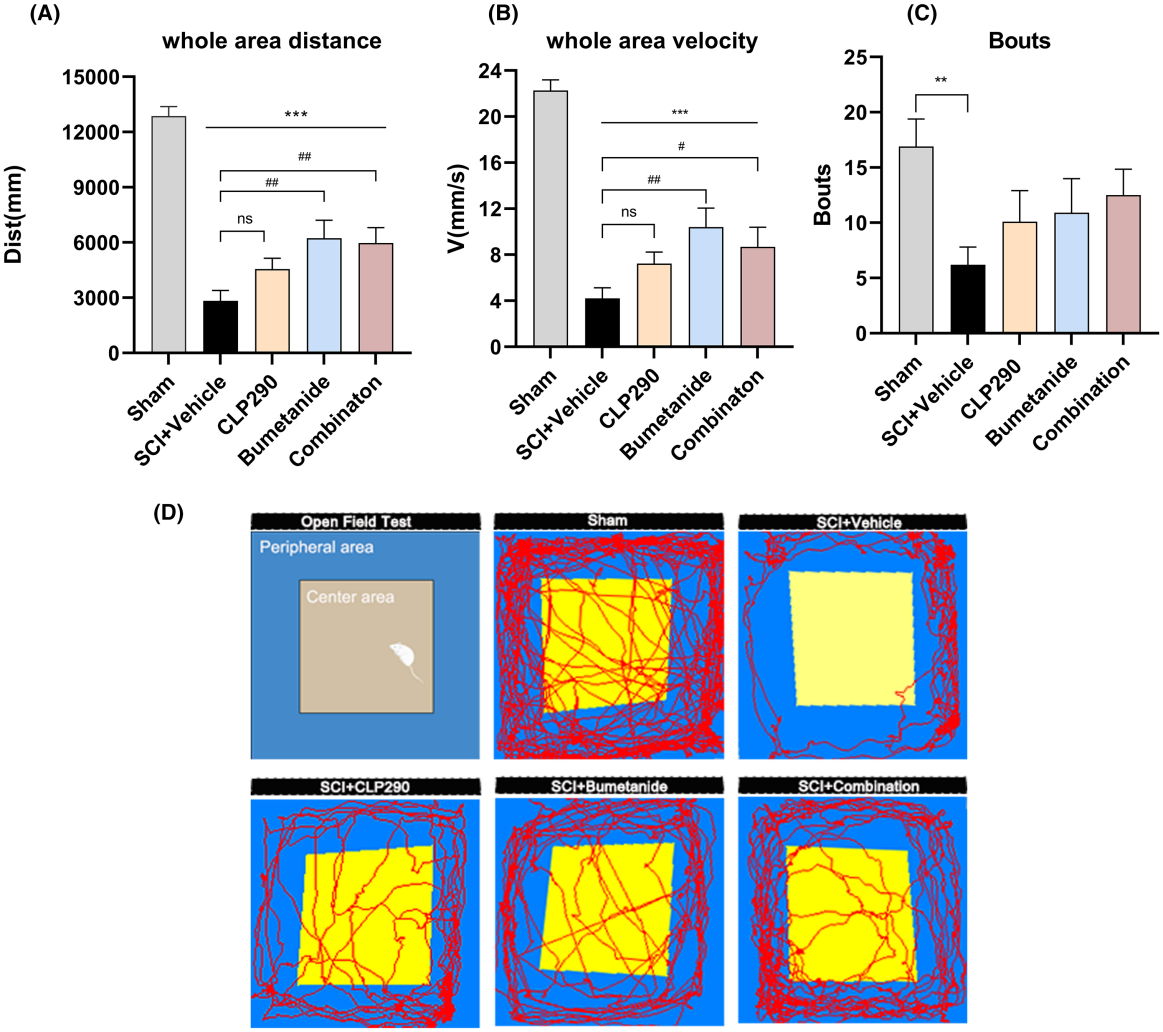
Effects of CLP290 and bumetanide, alone or in combination, on improving anxiety, depression, and activity levels following SCI. (A, B) Comparison of the total distance and average speed of horizontal movement at 56 dpi in different experimental groups. (C) Comparison of the central area entries (Bouts) at 56 dpi in different experimental groups. (D) Representative movement trajectories of different experimental groups of rats in the open field test. **p < 0.01, ***p < 0.001 (four intervention groups vs. sham); #*p* < 0.05, ##*p* < 0.01 (three drug treatment groups vs. SCI + vehicle); one‐way ANOVA, Tukey post‐hoc test. Values are mean ± SEM, Sham, *n* = 8, SCI + different intervention groups, *n* = 12.

This study used the open‐field test to evaluate anxiety, depression, and motor function in rats treated for SCI (Figure 3). At 56 dpi, the number of central zone crossings was significantly lower in the SCI + vehicle group compared to the sham group (*p* < 0.01). The other treatment groups showed improvement but did not reach statistical significance. At 56 dpi, the total distance of horizontal movement and mean velocity were significantly improved in the SCI + combination and SCI + bumetanide groups compared to the SCI + vehicle group (SCI + combination: distance, *p* < 0.01; velocity, *p* < 0.05; SCI + bumetanide: distance, *p* < 0.01; velocity, *p* < 0.01), whereas the SCI + CLP290 group showed slight improvement that was not statistically significant. The combination and bumetanide groups showed the greatest significant improvement in motor function, anxiety, and depression compared to the vehicle group at 56 dpi, although this improvement still lagged behind the scores in the sham group.

### 3.5 Effects of CLP290 and bumetanide, Alone or in Combination, on GABA_A_ receptor‐mediated inhibition function in the spinal cord

**FIGURE 4:**
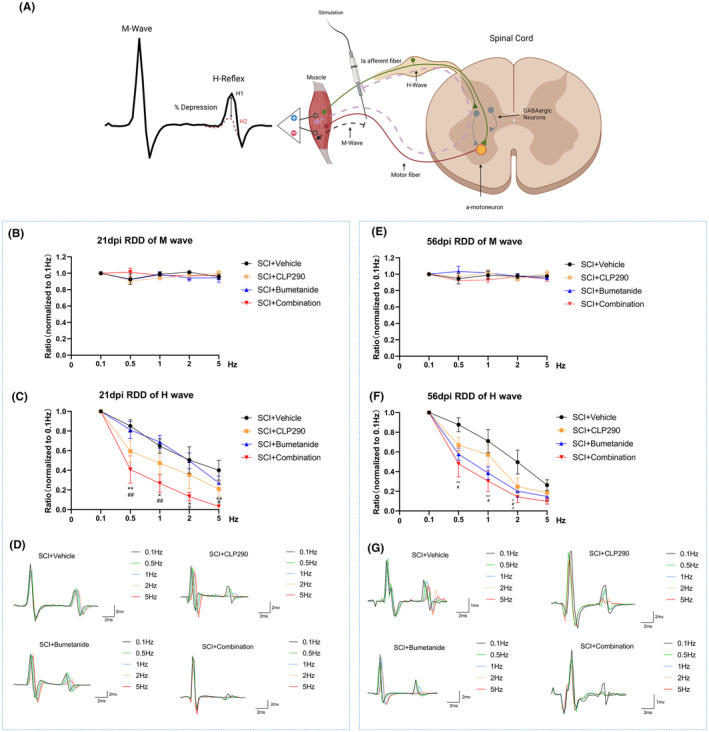
Effects of CLP290 and bumetanide, alone or in combination, on H‐reflex RDD levels. (A) Schematic diagram of the rate‐dependent depression (RDD) of the H‐reflex in the spinal cord. Under continuous electrical stimulation, the amplitude of the spinal cord Hoffman reflex gradually decreases with an increase in stimulation frequency. The % depression is calculated as the change in amplitude of the second recorded H‐reflex (H2) compared to the first in the train (H1). The M wave is generated by the orthodromic propagation of the stimulus along motor fibers. In contrast, the H‐reflex is a monosynaptic reflex transmitted by Ia afferent fibers and modulated by inhibitory interneurons (GABAergic neurons) in the spinal cord. (B, E) The RDD of the M wave in the experimental groups is shown with different stimulation rates at 21 and 56 dpi. (C) The RDD of the H wave in the experimental groups at 21 dpi. *p < 0.05, **p < 0.01 for SCI + combination versus SCI + vehicle; #*p* < 0.05, ##*p* < 0.01 for SCI + combination versus SCI + bumetanide. (F) The RDD of the H wave in the experimental groups at 56 dpi. *p < 0.05, **p < 0.01 for SCI + combination versus SCI + vehicle; #*p* < 0.05 for SCI + bumetanide versus SCI + vehicle; ^*p* < 0.05 for SCI + CLP290 versus SCI + vehicle. (D, G) Trajectories of H‐reflexes evoked by stimulation of the tibial nerve at 0.1 Hz (black), 0.5 Hz (green), 1 Hz (blue), 2 Hz (orange), and 5 Hz (red). Repeated measures two‐way ANOVA with Student Neuman‐Keuls post‐hoc test. Values are mean ± SEM, *n* = 8/group.

The schematic diagram of the rate‐dependent depression (RDD) of the H‐reflex is a reflection of the spinal cord inhibitory function, which is mediated mainly by GABA_A_R.^38^, ^39^, ^40^ The M‐wave which is an output of direct stimulation of motor neuronal axons does not altered upon the test (*p* > 0.05) (Figure 4B,E). In the present study, we aimed to compare RDD levels of H wave among different treatment groups to determine the recovery of GABA_A_R‐mediated inhibition of spinal cord neurons.

At 21 dpi, the combination treatment group demonstrated significantly higher RDD compared to the vehicle group (0.5 Hz: *p* = 0.009, 1 Hz: *p* = 0.01, 2 Hz: *p* = 0.032, 5 Hz: *p* = 0.002) and the bumetanide group (0.5 Hz: *p* = 0.011, 1 Hz: *p* = 0.003, 2 Hz: *p* = 0.025, 5 Hz: *p* = 0.027), while there was no statistically significant difference between the combination and the CLP290 group (*p* > 0.05) (Figure 4C,D); At 56 dpi, the RDD levels in the SCI + combination group were significantly higher than that in the SCI + vehicle group at 0.5, 1, and 2 Hz (0.5 Hz: *p* = 0.005, 1 Hz: *p* = 0.008, 2 Hz: *p* = 0.002) and not significantly different at 5 Hz (*p* = 0.064). At any frequency of electrical stimulation, there were no significant differences in RDD levels between the SCI + combination group and the other two monotherapy groups (*p* > 0.05). The RDD level in the SCI + bumetanide group was significantly higher than that in the SCI + vehicle group at 0.5, 1, and 2 Hz (0.5 Hz: *p* = 0.028, 1 Hz: *p* = 0.031, 2 Hz: *p* = 0.017) but not significantly different at 5 Hz (*p* = 0.187). The RDD levels in the SCI + CLP290 group compared with the SCI + vehicle group were significantly different only at a frequency of 2 Hz (*p* = 0.026) and not at any other frequency of electrical stimulation (*p* > 0.05) (Figure 4E,F).

Based on the comparison of RDD among different groups at 21 and 56 dpi, we found that the combination treatment group exhibited the most significant improvement in restoring RDD levels and GABA_A_R‐mediated inhibition, which was associated with alleviation of neuropathic pain.

### 3.6 Effects of CLP290 and bumetanide, Alone or in Combination, on KCC2 and NKCC1 protein expression following SCI

**FIGURE 5:**
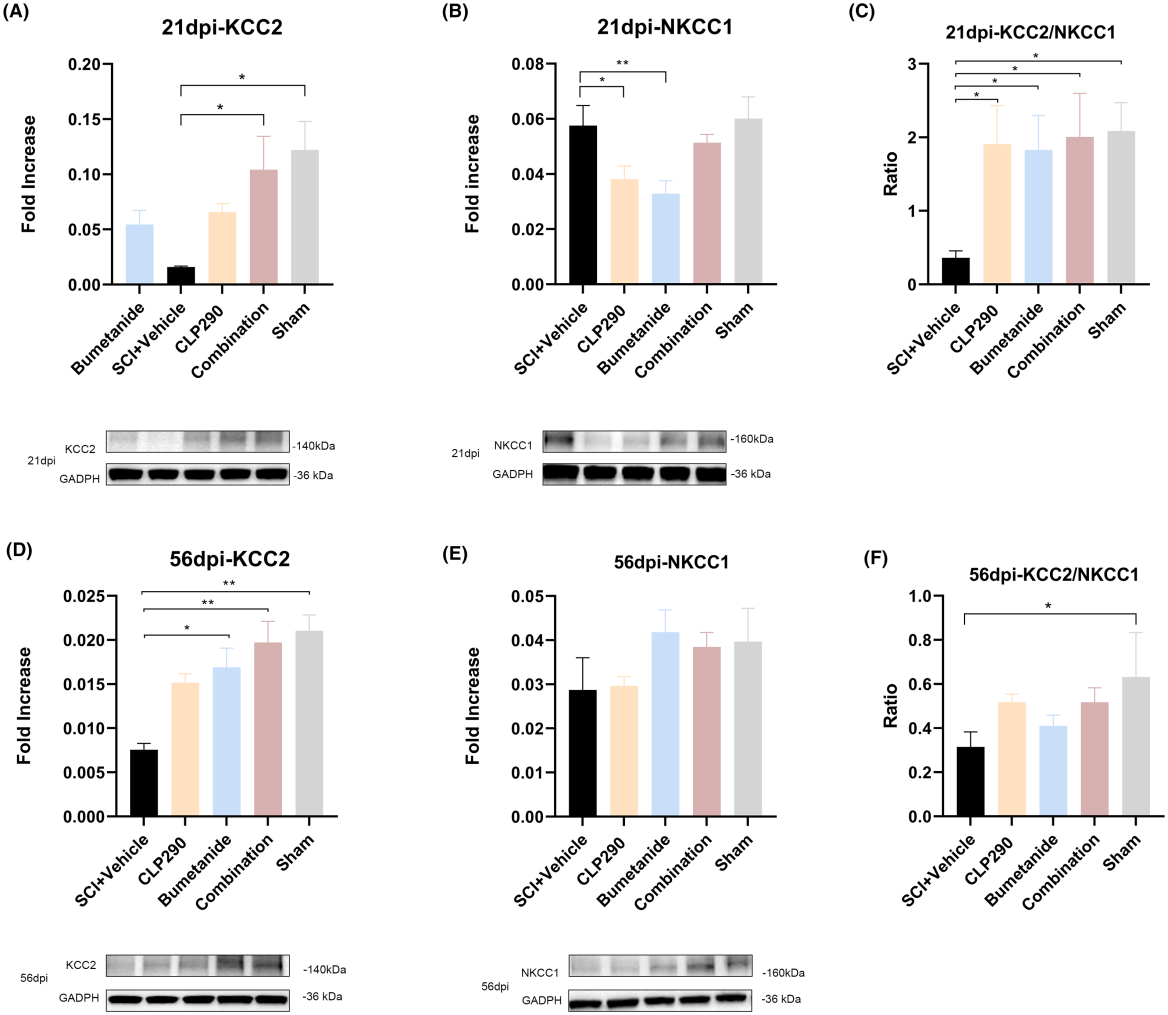
Effects of CLP290 and bumetanide, alone or in combination, on KCC2 and NKCC1 protein expression following SCI at 21 and 56 dpi. (A) Expression of KCC2 in the lumbar enlargement of the spinal cord in different experimental groups at 21 dpi. (B) The expression of NKCC1 in the lumbar enlargement of the spinal cord in different experimental groups at 21 dpi. (C) The KCC2/NKCC1 ratio in different experimental groups at 21 dpi. (D) Expression of KCC2 in the lumbar enlargement of the spinal cord in different experimental groups at 56 dpi. (E) The expression of NKCC1 in the lumbar enlargement of the spinal cord in different experimental groups at 56 dpi. (F) The KCC2/NKCC1 ratio in different experimental groups at 56 dpi. *p < 0.05 (different treatment groups or sham vs SCI + vehicle). One‐way ANOVA, Tukey post‐hoc test. Values are mean ± SEM, *n* = 4/group.

In this study, 21 and 56 dpi time points were selected to compare the expression of KCC2 and NKCC1 proteins in the lumbar enlargement of different intervention groups (Figure 5). At both 21 and 56 dpi, the level of KCC2 in the combined treatment group showed the greatest increase in protein expression that was significantly different from that of the SCI + vehicle group (21 dpi, *p* = 0.049; 56 dpi, *p* = 0.001), but did not reach statistical difference compared with that in the other two monotherapy groups. At 21 dpi, the expression level of the KCC2 protein in the other two monotherapy groups was slightly higher than that in the SCI + vehicle group, but this increase did not reach a statistical difference (*p* > 0.05). However, at 56 dpi, KCC2 protein in the SCI + bumetanide group showed significant difference compared to the SCI + vehicle group (*p* = 0.015), while there was no significant statistical difference in KCC2 expression between the SCI + CLP290 group and the SCI + vehicle group (*p* = 0.066). NKCC1 expression levels were significantly down regulated in the SCI + CLP290 and SCI + bumetanide groups compared with the SCI + vehicle group at 21dpi (CLP290 group: *p* = 0.033; bumetanide group: *p* = 0.009), while NKCC1 expression tended to decrease in the SCI + combination group, but no statistical differences were reached (*p* > 0.05).

The ratio of KCC2/NKCC1 was significantly higher in the three treatment groups than in the SCI + vehicle group at 21 dpi (CLP290: *p* = 0.027; bumetanide: *p* = 0.035; combination: *p* = 0.02; vs. SCI + vehicle). At 56 dpi, the KCC2/NKCC1 ratio in the three treatment groups was higher than the SCI + vehicle group, but no statistical differences were reached (*p* > 0.05).

### 3.7 Effects of CLP290 and bumetanide, Alone or in Combination, on KCC2 and NKCC1 immunofluorescence intensity following SCI

**FIGURE 6:**
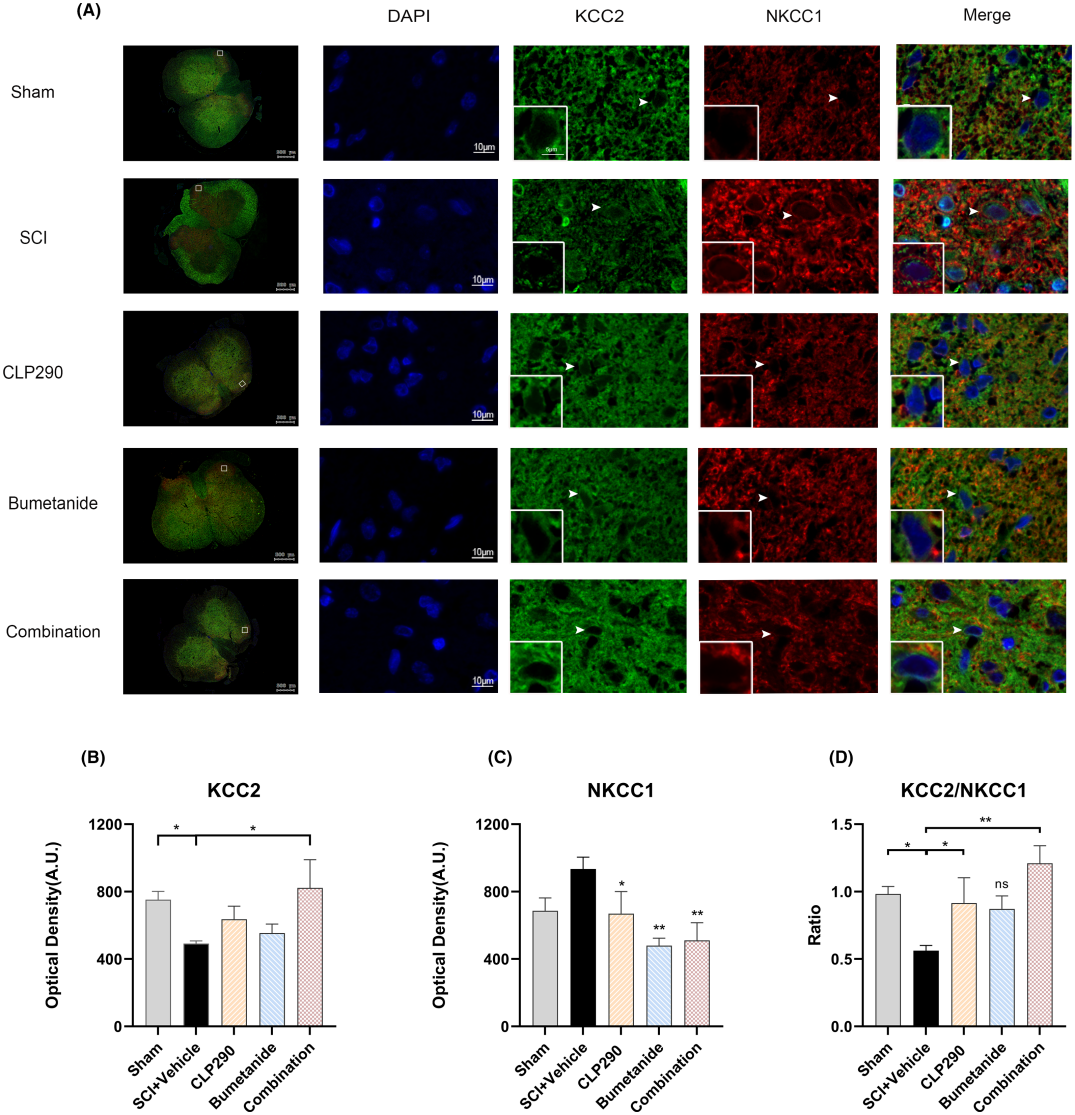
Effects of CLP290 and bumetanide, alone or in combination, on KCC2 and NKCC1 immunofluorescence intensity following SCI at 21 dpi. (A)The expression and distribution of KCC2 and NKCC1 in the dorsal horn of the lumbar enlargement after SCI was visualized by immunofluorescence staining. KCC2 protein expression was found to be uniform and intact on neuronal membranes in the sham group and in the three different drug treatment groups but not in the SCI + vehicle. In the latter group, KCC2 immunofluorescence distribution on neuronal membranes was discontinuous, and dot‐like internalization of KCC2 was observed in the cytoplasm. Conversely, NKCC1 protein expression was expressed evenly and completely on the neuronal cell membrane in the SCI + vehicle but less intensively and discontinuously in the sham and other three treatment groups. (B) Mean value of KCC2 fluorescence intensity in the dorsal horn of the lumbar enlargement in different experimental groups at 21 dpi. (C) Mean value of NKCC1 fluorescence intensity in the dorsal horn of the lumbar enlargement in different experimental groups at 21 dpi. (D) The ratio of KCC2/NKCC1 fluorescence intensity in different experimental groups at 21 dpi: *p < 0.05, **p < 0.01 (different treatment groups or sham vs SCI + vehicle). One‐way ANOVA, Tukey post‐hoc test. Values are mean ± SEM, *n* = 4/group.

The expression and distribution of KCC2 and NKCC1 in the dorsal horn of the lumbar enlargement after SCI was visualized by immunofluorescence staining. KCC2 protein expression was found to be uniform and intact on neuronal membranes in the sham group and in the three different drug treatment groups but not in the SCI + vehicle group. In the latter group, KCC2 immunofluorescence distribution on neuronal membranes was discontinuous, and dot‐like internalization of KCC2 was observed in the cytoplasm. Conversely, NKCC1 protein expression was expressed evenly and completely on the neuronal cell membrane in the SCI + vehicle group but less intensively and discontinuously in the sham and other three treatment groups (Figure 6A).

Further analysis of immunofluorescence intensity (Figure 6B–D) showed that at 21 dpi, the intensity of KCC2 up‐regulation was greatest in the combined treatment group, which was significantly different from the SCI + vehicle group (*p* = 0.014). The intensity of KCC2 immunization in the other two monotherapy groups did not reach statistical significance when compared with the SCI + vehicle group. The immunofluorescence intensity of NKCC1 in the three treatment groups was significantly down regulated when compared with the SCI + vehicle group (CLP290: *p* = 0.048, bumetanide: *p* = 0.002, and combination: *p* = 0.003). The KCC2/NKCC1 ratio in the SCI + vehicle group was notably reduced compared to the sham group (*p* = 0.016). Conversely, the KCC2/NKCC1 ratio was significantly elevated in both the SCI + CLP290 and SCI + combination groups compared to the SCI + Vehicle group (CLP290: *p* = 0.04; combination: *p* = 0.001). Although the SCI + bumetanide group had a slightly higher ratio compared to the SCI + vehicle group, this difference did not reach statistical significance at 21 dpi (*p* = 0.068).

## 4 DISCUSSION

### 4.1 The combination treatment provides superior results in improving neuropathic pain compared to treatment with a single medication

This study found that either CLP290 (50 mg/kg/day) or bumetanide (30 mg/kg/day), or the combination of the two drugs, can effectively improve neuropathic pain, anxiety, depression, and motor function in SCI, with the combination group showing the greatest improvement.

In various pathophysiological models involving reduced KCC2 activity, such as SCI, bilateral hemisection of the spinal cord, traumatic brain injury, PNI, and pressure models, administration of KCC2 agonist CLP290 has been found to significantly increase KCC2 activity and/or membrane expression and improve neuropathic pain, spasticity, and motor function.^22^, ^41^, ^42^, ^43^, ^44^, ^45^, ^46^ Specifically, Gagnon et al. demonstrated that CLP290 normalizes stimulus‐evoked responses in spinal nociceptive pathways that had been previously sensitized by nerve injury and reduced hypersensitivity. CLP290 was also found to reverse nociceptive sensory thresholds in morphine‐induced hyperalgesic animals.^30^ The NKCC1 inhibition bumetanide has also been shown to be effective in improving neuropathic pain in different models, including SCI, PNI, and traumatic brain injury models.^24^, ^47^, ^48^, ^49^, ^50^ Although CLP290 or bumetanide alone has previously been shown to be effective in improving neuropathic pain, there are no reports of their combined effects. In vivo, studies have shown that bumetanide is poorly permeable to the central nervous system after systemic administration.^51^ It selectively inhibits NKCCs without blocking other K–Cl cotransporters at low micromolar concentrations.^52^ Therefore, when combined with CLP290, bumetanide does not interfere with its effect on activating the KCC2. This study is the first to combine two chloride‐channel targeted drugs to treat neuropathic pain. It confirms that the combination treatment demonstrates the most pronounced efficacy, thus highlighting its substantial potential for the treatment of neuropathic pain in clinical practice.

### 4.2 Administration of CLP290, bumetanide, or a combination of both drugs effectively increased the ratio of KCC2/NKCC1, restored RDD levels, and enhanced GABA_A_R‐mediated inhibitory function in the spinal cord

Western blotting and immunofluorescence analysis results showed that all treatment groups were able to up‐regulate KCC2 expression and down‐regulate NKCC1 expression, thus increasing KCC2/NKCC1 ratio to varying degrees. The combination treatment was found to be the most effective in this regard. After treatment with the three drugs, an increase in the fluorescence intensity of KCC2 and a decrease in its cytoplasmic internalization on the neuronal cell membrane were observed. NKCC1 expression and localization exhibited the opposite trend.

The inhibition of the H‐reflex in a frequency‐dependent manner is an electrophysiological phenomenon that reflects spinal cord inhibitory function mediated by normal GABA_A_R. Previous studies have shown that RDD is impaired in neuropathic pain rat models and is associated with increases in spinal dorsal horn excitability mediated by GABA_A_R.^39^ In this study, we found that the improvement in neuropathic pain symptoms was closely related to the degree of restoration of RDD. Among the various treatment groups, the combination group exhibited the greatest recovery of RDD and the most significant improvement in neuropathic pain symptoms. This suggests that the amelioration of neuropathic pain is closely related to the recovery of GABA_A_R‐mediated inhibition in the spinal cord following SCI.

It has previously well been established that normal GABA neurotransmission depends on precise regulation of chloride levels in neural cells by KCC2 and NKCC1 proteins.^16^, ^17^, ^18^, ^19^ Thus, we implied that the combined use of the CLP290 and bumetanide resulted in a significant increase in the KCC2/NKCC1 ratio, driving [Cl^−^]_i_ below its E_Cl_, thereby restoring GABA_A_R‐mediated inhibitory function in the spinal cord, and relieving neuropathic pain symptoms.

### 4.3 Bumetanide significantly improves neuropathic pain in the long term, whereas CLP290 demonstrates a notable short‐term effect

In this study, we observed an intriguing phenomenon: The bumetanide group did not exhibit significant recovery in thermal hyperalgesia or RDD level at 21 dpi, but showed significant improvements at 56 dpi compared to SCI + vehicle group. Conversely, the short‐term effect of CLP290 alone was significant, but its long‐term pain‐relieving effect was not noteworthy. The above behavioral results are consistent with the protein molecular results. The exact mechanisms behind this phenomenon are not yet clear and need further investigation.

Nevertheless, the study still faces limitations that need to be addressed. Despite the combined CLP290 and bumetanide showing promising therapeutic effects, the synergistic or additive effects of these drugs remain to be elucidated. Furthermore, while this study primarily concentrated on neuropathic pain symptoms in rats with SCI, spasms were also observed in some rats. Both symptoms may have a common pathogenic mechanism to increase the spinal cord excitability mediated by GABA_A_R but through different cell types, which could be further studied.

## 5 CONCLUSION

Our findings indicate that strategies involving the use of CLP290, bumetanide, or a combination of these drugs can effectively increase the KCC2/NKCC1 ratio, restore chloride homeostasis, enhance spinal cord GABA_A_R‐mediated inhibitory function, and improve neuropathic pain following SCI. Bumetanide significantly improves neuropathic pain in the long term, whereas CLP290 demonstrates a notable short‐term effect. This study provides a foundation for the use of indirect ion‐targeted drug combinations to treat neuropathic pain following SCI and has significant clinical value and significance.

## AUTHOR CONTRIBUTIONS

JJL, FG, and YZP conceived and designed the study. JJL, FG, YZP, ZT, XXW, HK, CJZ, XX, DGY, LJD, and YY performed the experiments. YZP and XXW analyzed the data. YZP and ZT performed the mechanism drawing. YZP, JJL, and FG wrote the manuscript. All authors reviewed and approved the final manuscript.

## CONFLICT OF INTEREST STATEMENT

The authors declare no conflict of interest.

## Supporting information


**Figure S1.** The time‐course of KCC2 and NKCC1 protein expression in the lumbar enlargement following spinal cord injury.
**Figure S2.** Line chart showing weight changes over time, coupled with bar chart for direct group comparisons: A comprehensive view of rat body weight variations across experimental groups.

**Table S1.** Comparison of the statistical results of mechanical threshold among five experimental groups.

**Table S2.** Comparison of the statistical results of paw withdraw thermal latency among five experimental groups.

**Table S3.** Comparison of the statistical results of cold allodynia responses (%) among five experimental groups.

**Table S4.** Comparison of the statistical results of weight among five experimental groups (± *s*, g).

## Data Availability

The data that support the findings of this study are available from the corresponding author upon reasonable request.
