# Supplementary material for: Combined use of CLP290 and bumetanide alleviates neuropathic pain and its mechanism after spinal cord injury in rats
Source: CNS Neurosci Ther. 2024 Sep 12;30(9):e70045. doi: 10.1111/cns.70045 (PMC11393004; doi:10.1111/cns.70045)
Supplement: Supplementary file 1 — Figure S1. The time‐course of KCC2 and NKCC1 protein expression in the lumbar enlargement following spinal cord injury. Figure S2. Line chart showing weight changes over time, coupled with bar chart for direct group comparisons: A comprehensive view of rat body weight variations across experimental groups. [file CNS-30-e70045-s002.docx]

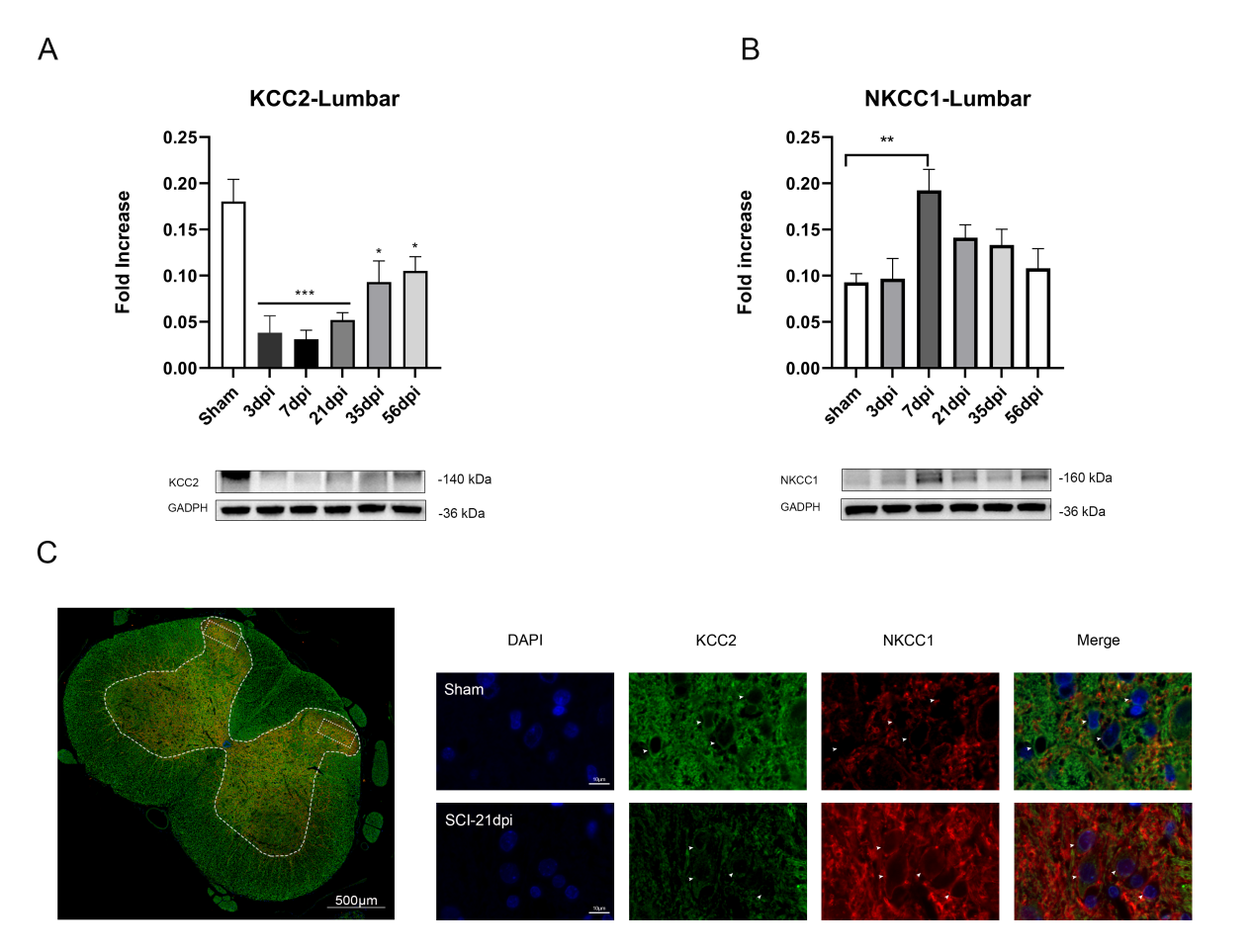


Supplementary Fig. 1 The time-course of KCC2 and NKCC1 protein expression in the lumbar enlargement following spinal cord injury. (A) Analysis of KCC2 and NKCC1 protein expression in the lumbar enlargement at 3, 7, 21, 35, and 56 days post-injury (dpi) using Western blotting. The expression of KCC2 protein was significantly decreased at 3, 7, 21, 35 and 56 dpi compared to the sham group, reaching the lowest level at 7 dpi (3, 7, 21 dpi: ****P*<0.001; 35,56 dpi: ***P*<0.05), followed by a gradual recovery trend. Values are mean ± s.e.m., n =4 / group. One-way ANOVA, Tukey post-hoc test. (B) NKCC1 protein expression was significantly increased at 7 dpi compared to the sham group (***P*<0.01 vs sham). At 3,21, 35, and 56 dpi, NKCC1 protein expression was slightly higher than in the sham group but did not reach statistical significance. One-way ANOVA, Tukey post-hoc test. (C) Immunolabeling with double staining of KCC2 and NKCC1. Confocal images of KCC2 and NKCC1 expression showed high and intact expression intensity of KCC2 protein on neuronal cell membranes in the dorsal horn of the lumbar enlargement in the sham group at 21 dpi, while the immunofluorescence intensity of KCC2 on neuronal cell membranes was low and discontinuous in the SCI group, with punctate and patchy internalization of KCC2 visible in the cytoplasm. Simultaneously, the immunofluorescence intensity of NKCC1 on neuronal cell membranes was low and discontinuous in the sham group, while the expression of NKCC1 on neuronal cell membranes in the SCI group showed strong and continuous immunofluorescence. Scale bar: 10μm. Left panel: Schematic diagram of the intact spinal cord lumbar enlargement, scale bar: 500μm.

Methods: Rats were randomly assigned to two groups for the induction of spinal cord injury (SCI) or sham surgery. The SCI group underwent spinal cord impact surgery at the T10 vertebral level with an impact force of 150 kyd. The sham group, serving as the normal control, underwent laminectomy at the T10 level (thoracic segment 10) without experiencing compression injury or receiving any pharmacological treatment. Following the procedures, protein immunoblotting was employed to describe the regulatory characteristics of NKCC1 and KCC2 protein expression at different time points in 24 rats (SCI group, with n=4 rats per time point at 3dpi, 7dpi, 21dpi, 35dpi, and 56dpi; Sham group: n=4 rats). Additionally, immunofluorescence analysis was performed on 8 rats at 21dpi, with 4 rats in each group.
