# Supplementary material for: Combined use of CLP290 and bumetanide alleviates neuropathic pain and its mechanism after spinal cord injury in rats
Source: CNS Neurosci Ther. 2024 Sep 12;30(9):e70045. doi: 10.1111/cns.70045 (PMC11393004; doi:10.1111/cns.70045)
Supplement: Supplementary file 2 — Table S1. Comparison of the statistical results of mechanical threshold among five experimental groups. [file CNS-30-e70045-s003.docx]

**Supplementary Table 1. Comparison of the Statistical Results of Mechanical Threshold among Five Experimental groups**

| Groups | Baseline | 7dpi | 21dpi | 35dpi | 56dpi |
| --- | --- | --- | --- | --- | --- |
| Sham (n=8) | 13.52±1.98Aa | 11.62±1.67Aa | 12.58±2.46Aa | 11.60±2.09Aa | 11.60±3.46Aa |
| SCI+ vehicle (n=12) | 12.40±3.85Aa | 0.94±0.52Bb | 0.47±0.16Cb | 1.38±1.76Cb | 1.66±1.29Db |
| SCI+CLP290 (n=12) | 13.65±2.84Aa | 0.76±0.68Bd | 3.36±2.45Bbc | 4.55±3.99Bbc | 5.56±3.04Cb |
| SCI + bumetanide (n=12) | 13.31±2.23Aa | 1.32±1.22Bbd | 2.61±1.5Bbcd | 3.85±3.11Bbc | 5.45±3.12Cb |
| SCI + combination (n=12) | 13.01±3.18Aa | 2.00±2.80Bd | 2.85±1.10Bd | 4.64±3.09Bc | 8.33±4.00Bb |
| F group/time/time*group | 44.14/140.86/6.34 | | | | |
| P group/time/time*group | <0.001/<0.001/<0.001 | | | | |

Footnotes: Values are mean ± S.E.M. Different capital letters indicate significant differences in statistical comparisons between groups (P < 0.05), while different lowercase letters indicate significant differences in statistical comparisons within groups (P < 0.05).
