# Supplementary material for: Combined use of CLP290 and bumetanide alleviates neuropathic pain and its mechanism after spinal cord injury in rats
Source: CNS Neurosci Ther. 2024 Sep 12;30(9):e70045. doi: 10.1111/cns.70045 (PMC11393004; doi:10.1111/cns.70045)
Supplement: Supplementary file 3 — Table S2. Comparison of the statistical results of paw withdraw thermal latency among five experimental groups. [file CNS-30-e70045-s004.docx]

**Supplementary Table 2. Comparison of the Statistical Results of Paw Withdraw Thermal Latency among Five Experimental groups**

| Groups | Baseline | 7dpi | 21dpi | 35dpi | 56dpi |
| --- | --- | --- | --- | --- | --- |
| Sham (n=8) | 14.10±1.56Aa | 13.89±2.03Aa | 14.10±2.24Aa | 14.64±1.15Aa | 15.09±1.81Aa |
| SCI+ vehicle (n=12) | 13.72±0.98Aa | 7.65±1.99Bc | 10.09±1.31BCb | 10.84±1.96ABCb | 10.52±2.21Bb |
| SCI+CLP290 (n=12) | 13.72±1.23Aa | 8.99±2.95Bb | 12.97±2.42Ba | 12.41±2.49ABCa | 12.11±1.91Ba |
| SCI + bumetanide (n=12) | 14.75±1.29Aa | 7.41±1.87Bac | 11.87±2.14BCb | 12.30±2.01ABCb | 14.21±1.85Aa |
| SCI + combination (n=12) | 14.61±2.39Aa | 9.07±1.55Bc | 12.59±2.68Bb | 13.25±2.19ABb | 15.63±2.19Aa |
| F group/time/time*group | 13.09/48.92/3.97 | | | | |
| P group/time/time*group | <0.001/<0.001/<0.001 | | | | |

Footnotes: Values are mean ± S.E.M. Different capital letters indicate significant differences in statistical comparisons between groups (P < 0.05), while different lowercase letters indicate significant differences in statistical comparisons within groups (P < 0.05).
