# Supplementary material for: Combined use of CLP290 and bumetanide alleviates neuropathic pain and its mechanism after spinal cord injury in rats
Source: CNS Neurosci Ther. 2024 Sep 12;30(9):e70045. doi: 10.1111/cns.70045 (PMC11393004; doi:10.1111/cns.70045)
Supplement: Supplementary file 4 — Table S3. Comparison of the statistical results of cold allodynia responses (%) among five experimental groups. [file CNS-30-e70045-s005.docx]

**Supplementary Table 3. Comparison of the Statistical Results of Cold Allodynia responses (%) among Five Experimental groups**

| Groups | Baseline | 7dpi | 21dpi | 35dpi | 56dpi |
| --- | --- | --- | --- | --- | --- |
| Sham (n=8) | 10.00±6.32Aa | 5.00±5.48Ba | 3.33±5.16C a | 10.00±6.32Ca | 8.33±9.83BCa |
| SCI+ vehicle (n=12) | 15.83±14.43Ab | 88.33±19.46Aa | 79.17±34.5Aa | 75.00±26.11Aa | 66.67±30.55Aa |
| SCI+CLP290 (n=12) | 8.33±10.3Ae | 95.83±11.65Aa | 68.33±23.29ABb | 60.00±26.63ABc | 44.17±18.32Bd |
| SCI+ bumetanide (n=12) | 7.50±12.15Ad | 91.67±11.15Aa | 60.83±25.03ABb | 60.83±19.75ABb | 40.00±22.96Bc |
| SCI+ combination (n=12) | 10.83±13.79Ad | 84.17±19.27Aa | 55.83±29.06ABb | 42.50±26.33ABbc | 30.00±28.28BCbc |
| F group/time/time*group | 22.23/46.00/196.00 | | | | |
| P group/time/time*group | <0.001/<0.001/<0.001 | | | | |

Footnotes: Values are mean ± S.E.M. Different capital letters indicate significant differences in statistical comparisons between groups (P < 0.05), while different lowercase letters indicate significant differences in statistical comparisons within groups (P < 0.05).
