# Supplementary material for: Combined use of CLP290 and bumetanide alleviates neuropathic pain and its mechanism after spinal cord injury in rats
Source: CNS Neurosci Ther. 2024 Sep 12;30(9):e70045. doi: 10.1111/cns.70045 (PMC11393004; doi:10.1111/cns.70045)
Supplement: Supplementary file 5 — Table S4. Comparison of the statistical results of weight among five experimental groups (± s, g). [file CNS-30-e70045-s001.docx]

**Temporal Changes and Inter-Group Differences in Rat Body Weight in Five Experimental Groups**

This study investigated temporal changes in rat body weight within experimental groups and compared these changes between groups. The results of intragroup comparisons showed that in the sham group, body weight gradually increased with time (comparisons between 7dpi and 0dpi, 21dpi and 7dpi, 35dpi and 21dpi, as well as 56dpi and 35dpi, P<0.05). In contrast, compared with 0dpi, the body weight of rats in the SCI+ different intervention groups significantly decreased at 7dpi (P<0.05), and then gradually increased significantly over time in these four different intervention groups (comparisons between 21dpi and 7dpi, 35dpi and 21dpi, as well as 56dpi and 35dpi, P<0.05). Intergroup comparisons revealed that there was no statistically significant difference in body weight between the five experimental groups at 0dpi (P>0.05). However, at 7dpi, 21dpi, 35dpi, and 56dpi, the body weight of the sham group was significantly higher than that of the SCI+ different intervention groups (P<0.05).

Supplementary Table 4. Comparison of the Statistical Results of Weight among Five Experimental groups ($\bar{x}$±*s*，g)

| Groups | Baseline | 7dpi | 21dpi | 35dpi | 56dpi |
| --- | --- | --- | --- | --- | --- |
| SCI +Vehicle (*n*=12) | 213.75±10.96Ac | 204.67±8.67Bd | 220.67±13.71Cc | 253.50±21.50Bb | 278.25±18.15Ba |
| SCI +Bumetanide (*n*=12) | 216.83±4.30Ad | 207.17±8.38Be | 226.83±13.55BCc | 247.92±24.89Bb | 277.75±29.02Ba |
| SCI+CLP290 (*n*=12) | 213.00±7.16Ad | 203.19±12.41Be | 222.25±12.69Cc | 249.75±16.24Bb | 274.92±18.05Ba |
| SCI+Combination (*n*=12) | 218.50±10.48Ad | 210.33±12.97Be | 237.92±15.20Bc | 261.25±18.69Bb | 283.58±20.83Ba |
| Sham (*n*=8) | 211.63±5.29Ae | 225.50±8.94Ad | 258.75±14.12Ac | 294.00±11.76Ab | 320.88±8.27Aa |
| *F* group/time/time*group | 11.85/370.90/4.50 | | | | |
| *P* group/time/time*group | <0.001/<0.001/<0.001 | | | | |

Footnote: A two-way repeated measures ANOVA was employed, with SNK tests for post-hoc analysis. Significant differences between groups are indicated by different uppercase letters (*P*<0.05), while significant differences within groups are denoted by distinct lowercase letters (*P*<0.05).

(A)

(B)


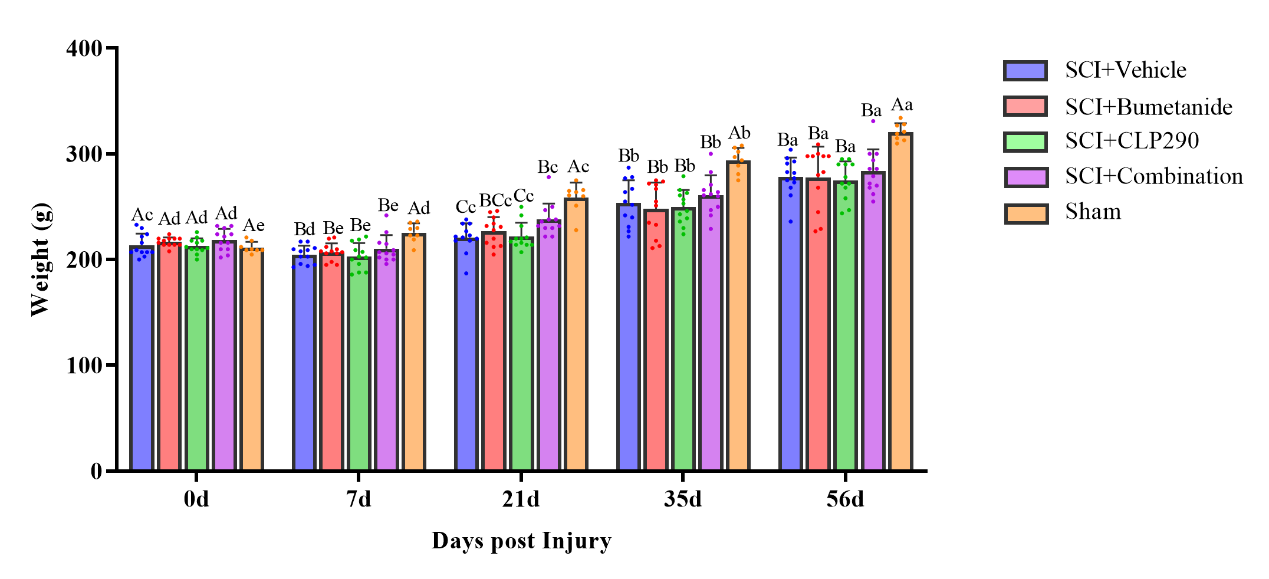


**Supplementary Fig. 2: Line Chart Showing Weight Changes Over Time, Coupled with Bar Chart for Direct Group Comparisons: A Comprehensive View of Rat Body Weight Variations Across Experimental Groups**

A two-way repeated measures ANOVA was employed, with SNK tests for post-hoc analysis. Significant differences between groups are indicated by different uppercase letters (*P*<0.05), while significant differences within groups are denoted by distinct lowercase letters (*P*<0.05). (A)This line chart depicts the variations in body weight of rats from different experimental groups over time. Each line represents a distinct group, enabling a clear comparison of weight gain or loss trends among the groups. Significant changes in body weight were observed at different time points across the five treatment groups (*P*<0.05). Intra-group comparison: Within the Sham group, body weight gradually increased over time (*P*<0.05 for comparisons between 7 dpi vs. 0 dpi, 21 dpi vs. 7 dpi, 35 dpi vs. 21 dpi, and 56 dpi vs. 35 dpi). In contrast, the SCI +Vehicle, SCI +CLP290, SCI +Bumetanide, and SCI +Combination groups all showed significant decreases in body weight at 7 dpi compared to 0 dpi(*P*<0.05). After 7 dpi, the body weight in these four groups significantly increased over time (*P*<0.05 for comparisons between 21 dpi vs. 7 dpi, 35 dpi vs. 21 dpi, and 56 dpi vs. 35 dpi). (B) This bar chart compares the body weight of rats in different experimental groups at various time points. The chart provides a clear visualization of weight changes over time, allowing for a straightforward comparison among the groups. Inter-group comparison: At 0dpi, there was no statistically significant difference in body weight among the five experimental groups (*P*>0.05). However, from 7 dpi to 56 dpi, significant differences in body weight were observed among the five groups (*P*<0.05). At 7dpi, 21 dpi, 35 dpi and 56 dpi, the Sham group had significantly higher body weight compared to the SCI +Vehicle, SCI+CLP290, SCI +Bumetanide, and SCI +Combination groups (*P*<0.05).
